# Seasonal Variation in Serum Testosterone Levels: Evidence from 2 Large Institutional Databases

**DOI:** 10.5152/tud.2023.23077

**Published:** 2023-09-01

**Authors:** David Miller, Aaron Gurayah, Alexander Weber, Kyle Schuppe, Mohamadhusni Zarli, Alexandra Dullea, Kathleen Hwang, Ranjith Ramasamy

**Affiliations:** 1 Department of Urology, UPMC, Pennsylvania, USA; 2 Desai Sethi Urology Institute, University of Miami, Florida, USA; 3 Washington State University Elson S. Floyd College of Medicine, Washington, USA; 4 Nova Southeastern University College of Osteopathic Medicine, Florida,USA

**Keywords:** Seasons, testosterone, testosterone replacement therapy

## Abstract

**Objective:**

Seasonal variations in testosterone levels have been reported in some studies, but the results are inconsistent. In this study, we aimed to determine if clinically relevant seasonal variability in testosterone levels exists using a large cohort of men from 2 different institutions, 1 located in an area with seasons (Pittsburgh, Pa) and 1 without seasons (Miami, Fla).

**Methods:**

Using 2 institutional databases, testosterone levels were obtained for men ages 18-99 from 2010 to 2021 who had at least 2 morning testosterone levels drawn within a 2-year period. All samples were analyzed with liquid chromatography with tandem mass spectrometry. To avoid potential confounding by testosterone altering medications patients who were currently or previously on exogenous testosterone, endogenous testosterone-stimulating medications, testosterone-suppressing medications, and aromatase inhibitors were excluded from the study.

**Results:**

There were 9495 and 16 171 total testosterone levels measured from Miami and Pittsburgh, respectively, with all men having 2 or more levels. There was no statistically significant variation in testosterone levels for the overall cohort in Pittsburgh or Miami, respectively. Additionally, when stratified by age group, no individual groups were found to have significant seasonal variability.

**Conclusion:**

Our findings suggest that although there is differing total testosterone levels between men who reside in 2 different climates, there is no significant variability in testosterone levels between seasons. Therefore, testosterone levels can be checked and interpreted without the need to account for the season during which they were drawn.

**Main Points**

- There is no significant variability in testosterone levels between seasons.
- Interestingly, our study did show a statistically significant difference in mean total testosterone concentrations between Miami and Pittsburgh, with men in Miami having higher testosterone levels.
- Testosterone levels can be checked and interpreted without the need to account for the season during which they were drawn.

## Introduction

Awareness of testosterone deficiency and the benefits of initiating hormone replacement for men has increased over the past decade. The prevalence of low testosterone in men over 45 years old is reported to be around 39%.^1^ It is estimated that nearly 6.5 million men between the ages of 30 and 70 years will have symptomatic testosterone deficiency by the year 2025.^2^ Low testosterone levels have been shown to be associated with a wide range of issues including erectile dysfunction, cardiovascular comorbidities, diabetes mellitus, osteoporosis, emotional lability, and metabolic syndrome.^3^ Clinical manifestation of low testosterone may include sleep disturbance, low libido, subfertility, loss of body hair, anemia, reduced strength/physical performance, and increased body fat.^4^ Testosterone therapy (TT) can correct symptoms associated with testosterone deficiency. Testosterone therapy has the potential to improve libido, erectile function, anemia, increase bone mass density, improved glycemic control, and depressive mood.^5,6^

For accurate evaluation clinicians must consider the variations that testosterone undergoes in the human body.^7^ The diurnal variation of testosterone is well characterized; its levels peak in the morning and decline during the day.^8,9^ Thus, per American Urological Association guidelines, it is recommended to check morning levels of testosterone for accurate evaluations of testosterone deficiency.^10,11^ However, there is a lack of consensus on whether there are significant seasonal variations in serum testosterone levels. Several studies have reported possible peaks and troughs across seasons.^12-15^ Both Dabbs and Lee showed a peak of testosterone levels in winter, while Ballatsella et al and Zornitski et al reported that testosterone peaks in summer. Conversely, other studies concluded that there are no seasonal variations in testosterone.^16,17^

Our objective was to determine if there is clinically relevant seasonal variability in testosterone levels. We utilized retrospective data of serum testosterone levels in a large cohort of men from 2 different institutions, 1 located in an area with seasons (Pittsburgh, Pa) and 1 without seasons (Miami, Fla). We hypothesized that there would be significant seasonal variability for Pittsburgh, Pa, and that there would be no significant variability for Miami, Fla.

## Material and Methods

Using 2 institutional databases, testosterone levels were obtained for men ages 18-99 from 2010 to 2021 who had at least 2 morning testosterone levels drawn within a 2-year period for any reason. Seasons were defined as December 21–March 19 for winter, March 20–June 20 for spring, June 21–September 1 for summer, and September 2–December 20 for autumn. Patients were divided into age groups that were defined as men aged 18-40, 41-50, 51-60, 61-70, and over 70 years old. All samples were analyzed with liquid chromatography with tandem mass spectrometry (LC-MS/MS). To avoid potential confounding by testosterone altering medications patients who were currently or previously on exogenous testosterone, endogenous testosterone stimulating medications (clomiphene citrate and hCG), testosterone-suppressing medications [gonadotropin hormone-releasing hormone (GnRH) agonist and GnRH antagonist], and aromatase inhibitors were excluded from the study. This study was reviewed and approved by the institutional review board from the University of Miami and the University of Pittsburgh (22030010). Additionally, this study was deemed exempt from need to obtain informed consent as it was retrospective in nature. 

### Statistical analysis

*T*-tests were used to compare demographic variables between cities given their normal distributions. A panel regression model was used to evaluate differences in testosterone levels by season in each respective city. Panel regression is a statistical technique implemented to analyze the relationship of a series of independent variables collected in pooled cross-sectional observations over time with a dependent variable, and it has been used in longitudinal data analysis.^18^ This model controls for the effects of unbalanced and/or heterogeneous data (i.e., data collected on subjects at nonuniform time points with an uneven number of observations). A series of independent *t*-tests were used to compare variability of testosterone levels between age groups from each location. A *P*-value of .05 was considered statistically significant. All statistical analyses were performed with R version 4.1.1.

## Results

There were 9495 and 16 171 total testosterone levels measured from Miami and Pittsburgh respectively, with all patients having 2 or more levels. Miami patients were older on average than those from Pittsburgh (59 vs. 52 respectively). Additionally, patients from Miami had statistically higher overall testosterone levels than those from Pittsburgh (417 ng/dL vs. 397 ng/dL, *P* < .01; Table 1). This statistically significant difference between mean testosterone levels continued between the locations when stratifying by age group (*P* < 0.01). The median time between testosterone levels for the Pittsburgh cohort was 169 and for the Miami cohort it was 469 days. As expected, mean temperature and minutes of daylight for each city were variable between the 2 cities. The mean temperature difference between Miami and Pittsburgh was 36°F in the winter and this difference decreased to 11°F in the summer. There was no statistically significant seasonal variation in testosterone levels in Pittsburgh or Miami, for the overall cohorts respectively (Table 2). Patients had their lowest testosterone level during the summer in Pittsburgh ∆T of –2.65 ng/dL (*P* = .63). Patients had their lowest testosterone levels during Spring in Miami ∆T of –4.28 ng/dL (*P* = .47). However, these variations were not large enough to be considered statistically significant nor clinically relevant. When stratified by age group, there was no consistent peak or trough testosterone level by season across each age category. Furthermore, no age groups exhibited statistically significant seasonal variability (Table 3).

## Discussion

We present one of the largest cross-sectional studies to date that has been conducted to evaluate for seasonal changes in testosterone. The results of our study were contrary to the initial hypothesis, we report that men’s testosterone levels did not vary from season to season. This was the case regardless of age or whether the local climate experiences a high degree of seasonal variability as seen by the lack of significant seasonal testosterone fluctuations from patients tested in either Miami or Pittsburgh. Thus, our results support that there is no significant seasonal variation in testosterone levels.

Previous studies have demonstrated conflicting results, with some reporting variability having peaks of testosterone in warmer months and some having their testosterone peaks in colder months^12-15,19,20^ while others report no discernable variation from season to season.^16,17,21,22^ Although these studies have reported statistically significant differences in testosterone levels by season, this does not necessarily indicate clinical significance. Lee et al report that in their cross-sectional evaluation of middle aged policemen a peak-to-trough difference of 1.48ng/mL.^17^ Santi et al report an average peak of testosterone levels in the summer months of 5.44 ng/mL, with a trough in autumn where they found testosterone declined to an average of 5.26 ng/mL.S^19^ Another large study by Zornitzki found a difference between peak and trough of 1 nmol/L.^15^

Interestingly, our study did show a statistically significant difference in mean total testosterone concentrations between Miami and Pittsburgh. This is unusual in that there has long been a negative association between testosterone levels and age, the cohort of patients in Miami, despite being 7 years older on average, also had higher average levels of testosterone.^23^ Although this could be due to several confounding factors inherent to the participants such as the climate. Our finding is in agreement with a large study that showed increased testosterone levels correlated to higher environmental temperatures and longer daylight duration.^19^ Conversely, there are several studies that have reported higher average levels of testosterone in winter seasons.^13,14,20^ The study by Lee et al also showed that, when broken into quartiles by age, only the group of men of ages 48-57 showed statistically significant seasonal fluctuations in testosterone. In our study, we did not see any significant seasonal fluctuation when stratifying by age.

Notably, most studies on the seasonality of testosterone levels were conducted in only a few latitudes, with more northerly ones experiencing great fluctuations in overall daylight that could confound results in relation to the rest of the world. Two proposed hypotheses for seasonal fluctuations in testosterone are circadian changes in melatonin secretion and temperatures affecting Leydig cell activity, both of which have had evidence prior to suggested effects on steroidogenesis.^19,24,25^ However, hyperthermia has shown negative effects on steroidogenesis in rats, conflicting with theories that warmer temperatures were directly causing elevated testosterone levels.^24,25^ Additionally, melatonin administration over the short and long term has shown no effect on serum testosterone levels.^26,27^ The major melatonin metabolite 6-sulfatoxymelatonin has been connected to variable luteinizing hormone levels, but this effect did not extend to testosterone levels.^28^ Finally, variability in sleep across seasons does not appear to be a significant factor as total sleep duration did not change between winter and summer in prior studies looking at seasonal variation of testosterone.^28^

Our study must be interpreted in the context of several limitations. First, we did not assess a variety of potentially significant patient characteristics including ethnicity, body mass index, and medical comorbidities. Second, no information is available about free testosterone, gonadotropins, estradiol, and liver function for our cohort. Thus, we are not able to consider possible seasonal variation of bioavailable testosterone, testosterone-to-estrogen ratio, or relationship of testosterones with gonadotropins. Third, we did not account for individual exposure to environments outside of their local areas, thus some patients may have had a variable seasonal exposure due to travel. Fourth, there is variability in temperature and daylight hours for the locations that does not have a significant change of seasons (Miami, Fla), which may confound results.

Despite these weaknesses, our study has several strengths. We present findings of seasonal testosterone levels from the largest cohort of men reported thus far, living at 2 separate latitudes, over a long longitudinal time frame interval, without known testosterone-altering medications, and considered amounts of both daylight and local temperatures. Our findings suggest that although there are differing total testosterone levels between 2 different climates, there is no significant variability in testosterone levels between seasons. Therefore, testosterone levels can be checked and interpreted without the need to account for the season during which they were drawn.

## Figures and Tables

**Table 1.** Study Demographics

	Pittsburgh (N = 16 171)	Miami (N = 9495)	*P*
Age (M ± IQR) [years]^*^	52 ± 21	59 ± 20	<.01
Median time between testosterone measurements (days)	169	469	
Number of follow-up (μ)	2.4	2.8	<.01
Overall total testosterone (μ ± SD) [ng/dL]	397 ± 288	417 ± 242	<.01
Spring (μ ± SD)	398 ± 294	412 ± 246	
Summer (μ ± SD)	394 ± 256	417 ± 226	
Fall (μ ± SD)	401± 311	417 ± 237	
Winter (μ ± SD)	396 ± 295	422 ± 259	
Age			<.01
18-40	443 ± 260	418 ± 342	
41-50	446 ± 283	397 ± 305	
51-60	418 ± 249	403 ± 247	
61-70	408 ± 213	392 ± 251	
≥70	393 ± 219	366 ± 262	
Mean temperature (°F)			
Spring	51	78	
Summer	73	84	
Fall	55	75	
Winter	32	68	
Mean daylight (minutes)			
Spring	794	801	
Summer	872	780	
Fall	671	661	
Winter	592	682	

Winter: December 21–March 19, Spring: March 20–June 20, Summer: June 21–September 1, Autumn: September 2–December 20.

M, median; μ, mean.

^*^Based on age at first visit.

**Table 2.** Changes in Testosterone Levels by Season for the Entire Cohort

	Pittsburgh		Miami	
	Δ*T *(ng/dL)	*P*	Δ*T *(ng/dL)	*P*
Winter	Ref	–	Ref	–
Spring	1.63	0.78	−4.28	.47
Summer	−2.65	0.63	0.95	.87
Fall	5.63	0.34	1.71	.78

**Table 3.** Changes in Testosterone Levels by Season

	**Miami**		**Pittsburgh**	
	Δ*T* (ng/dL)	*P*	Δ*T *(ng/dL)	*P*
(18-40 years old)				
Winter	Ref	–	Ref	–
Spring	−13.02	0.46	14.75	.56
Summer	−11.54	0.51	14.09	.80
Fall	−20.51	0.26	14.89	.69
(41-50 years old)				
Winter	Ref	–	Ref	–
Spring	8.43	0.68	2.73	.85
Summer	5.17	0.79	−13.85	.32
Fall	23.73	0.25	−5.60	.70
(51-60 years old)				
Winter	Ref	–	Ref	–
Spring	−12.03	0.35	16.65	.08
Summer	−4.09	0.74	4.54	.62
Fall	0.34	0.98	12.61	.19
(61-70 years old)				
Winter	Ref	–	Ref	–
Spring	−3.87	0.72	−8.96	.43
Summer	−8.40	0.43	9.50	.38
Fall	0.85	0.94	11.91	.31
(≥70 years old)				
Winter	Ref	–	Ref	–
Spring	−3.22	0.73	−5.84	.68
Summer	13.67	0.13	−14.06	.30
Fall	0.09	0.99	10.43	.48

## References

[1] Mulligan T Frick MF Zuraw QC Stemhagen A Mcwhirter C . Prevalence of hypogonadism in males aged at least 45 years: the HIM study. Int J Clin Pract. 2006;60(7):762 769. (10.1111/j.1742-1241.2006.00992.x) 16846397 PMC1569444

[2] Araujo AB Esche GR Kupelian V , et al. Prevalence of symptomatic androgen deficiency in men. J Clin Endocrinol Metab. 2007;92(11):4241 4247. (10.1210/jc.2007-1245) 17698901

[3] Barbonetti A D’Andrea S Francavilla S . Testosterone replacement therapy. Andrology. 2020;8(6):1551 1566. (10.1111/andr.12774) 32068334

[4] Wu FCW Tajar A Pye SR , et al. Hypothalamic-pituitary-testicular axis disruptions in older men are differentially linked to age and modifiable risk factors: the European male aging study. J Clin Endocrinol Metab. 2008;93(7):2737 2745. (10.1210/jc.2007-1972) 18270261

[5] Heufelder AE Saad F Bunck MC Gooren L . Fifty-two-week treatment with diet and exercise plus transdermal testosterone reverses the metabolic syndrome and improves glycemic control in men with newly diagnosed Type 2 diabetes and subnormal plasma testosterone. J Androl. 2009;30(6):726 733. (10.2164/jandrol.108.007005) 19578132

[6] Snyder PJ Bhasin S Cunningham GR , et al. Effects of testosterone treatment in older men. N Engl J Med. 2016;374(7):611 624. (10.1056/NEJMoa1506119) 26886521 PMC5209754

[7] Lunenfeld B Mskhalaya G Zitzmann M , et al. Recommendations on the diagnosis, treatment and monitoring of hypogonadism in men. Aging Male. 2015;18(1):5 15. (10.3109/13685538.2015.1004049) 25657080 PMC4648196

[8] González-Sales M Barrière O Tremblay PO Nekka F Desrochers J Tanguay M . Modeling testosterone circadian rhythm in hypogonadal males: effect of age and circannual variations. AAPS J. 2016;18(1):217 227. (10.1208/s12248-015-9841-6) 26553482 PMC4706275

[9] Brambilla DJ O’Donnell AB Matsumoto AM McKinlay JB . Lack of seasonal variation in serum sex hormone levels in middle-aged to older men in the Boston area. J Clin Endocrinol Metab. 2007;92(11):4224 4229. (10.1210/jc.2007-1303) 17684044

[10] Mulhall JP Trost LW Brannigan RE , et al. Evaluation and management of testosterone deficiency: AUA Guideline [AUA guideline]. J Urol. 2018;200(2):423 432. (10.1016/j.juro.2018.03.115) 29601923

[11] Bhasin S Cunningham GR Hayes FJ , et al. Testosterone therapy in men with androgen deficiency syndromes: an Endocrine Society clinical practice guideline. J Clin Endocrinol Metab. 2010;95(6):2536 2559. (10.1210/jc.2009-2354) 20525905

[12] Bellastella A Criscuolo T Mango A Perrone L Sinisi AA Faggiano M . Circannual rhythms of plasma luteinizing hormone, follicle-stimulating hormone, testosterone, prolactin and cortisol IN prepuberty. Clin Endocrinol (Oxf). 1983;19(4):453 459. (10.1111/j.1365-2265.1983.tb00019.x) 6414746

[13] Dabbs JM . Age and seasonal variation in serum testosterone concentration among men. Chronobiol Int. 1990;7(3):245 249. (10.3109/07420529009056982) 2268886

[14] Lee JH Lee SW . Monthly variations in serum testosterone levels: results from testosterone screening of 8,367 middle-aged men. J Urol. 2021;205(5):1438 1443. (10.1097/JU.0000000000001546) 33350323

[15] Zornitzki T Tshori S Shefer G Mingelgrin S Levy C Knobler H . Seasonal variation of testosterone levels in a large cohort of men. Int J Endocrinol Bevilacqua A , et al., ed. 2022;2022:6093092. (10.1155/2022/6093092) 35782408 PMC9242810

[16] Abbaticchio G de Fini M Giagulli VA Santoro G Vendola G Giorgino R . Circannual rhythms in reproductive functions of human males, correlations among hormones and hormone-dependent parameters. Andrologia. 1987;19(3):353 361. (10.1111/j.1439-0272.1987.tb02314.x) 3115144

[17] Lee JH . Variation in levels of serum testosterone in monthly samples from healthy men. Eur Urol Open Sci. 2020;19:e130. (10.1016/S2666-1683(20)32629-X)

[18] Noh JW Kwon YD Park J Oh IH Kim J . Relationship between physical disability and depression by gender: A panel regression model. PLOS ONE Weng X , ed. 2016;11(11):e0166238. (10.1371/journal.pone.0166238) 27902709 PMC5130183

[19] Santi D Spaggiari G Granata ARM , et al. Seasonal changes of serum gonadotropins and testosterone in men revealed by a large data set of real-world observations over nine years. Front Endocrinol. 2019;10:914. (10.3389/fendo.2019.00914) PMC6965064 31998242

[20] Svartberg J Jorde R Sundsfjord J Bønaa KH Barrett-Connor E . Seasonal variation of testosterone and waist to hip ratio in men: the Tromsø study. J Clin Endocrinol Metab. 2003;88(7):3099 3104. (10.1210/jc.2002-021878) 12843149

[21] Svartberg J Barrett-Connor E . Could seasonal variation in testosterone levels in men be related to sleep? Aging Male. 2004;7(3):205 210. (10.1080/13685530412331284696) 15669539

[22] Moskovic DJ Eisenberg ML Lipshultz LI . Seasonal fluctuations in testosterone-estrogen ratio in men from the Southwest United States. J Androl. 2012;33(6):1298 1304. (10.2164/jandrol.112.016386) 22790643

[23] Travison TG Araujo AB Kupelian V O’Donnell AB McKinlay JB . The relative contributions of aging, health, and lifestyle factors to serum testosterone decline in men. J Clin Endocrinol Metab. 2007;92(2):549 555. (10.1210/jc.2006-1859) 17148559

[24] Kanter M Aktas C . Effects of scrotal hyperthermia on Leydig cells in long-term: a histological, immunohistochemical and ultrastructural study in rats. J Mol Histol. 2009;40(2):123 130. (10.1007/s10735-009-9222-5) 19484498

[25] Kim JH Park SJ Kim TS Kim JM Lee DS . Testosterone production by a Leydig tumor cell line is suppressed by hyperthermia-induced endoplasmic reticulum stress in mice. Life Sci. 2016;146:184 191. (10.1016/j.lfs.2015.12.042) 26739509

[26] Luboshitzky R Levi M Shen-Orr Z Blumenfeld Z Herer P Lavie P . ­Long-term melatonin administration does not alter pituitary-gonadal hormone secretion in normal men. Hum Reprod. 2000;15(1):60 65. (10.1093/humrep/15.1.60) 10611189

[27] Zizzo J Reddy R Kulkarni N Blachman-Braun R Ramasamy R . Impact of low-dose melatonin supplementation on testosterone levels in U.S. adult males. Urology. 2022;169:92 95. (10.1016/j.urology.2022.07.048) 35963395

[28] Ruhayel Y Malm G Haugen TB , et al. Seasonal variation in serum concentrations of reproductive hormones and urinary excretion of 6-sulfatoxymelatonin in men living north and south of the Arctic circle: a longitudinal study. Clin Endocrinol (Oxf). 2007;67(1):85 92. (10.1111/j.1365-2265.2007.02843.x) 17547693

